# Molnupiravir Does Not Induce Mutagenesis in Host Lung Cells during SARS-CoV-2 Treatment

**DOI:** 10.1177/11779322221085077

**Published:** 2022-03-23

**Authors:** John Maringa Githaka

**Affiliations:** Department of Biochemistry, University of Alberta, Edmonton, AB, Canada

**Keywords:** Molnupiravir, mutagenesis, SARS-CoV-2, COVID-19, antiviral drug

## Abstract

As SARS-CoV-2 continues to evolve and spread with the emergence of new variants, interest in small molecules with broad-spectrum antiviral activity has grown. One such molecule, Molnupiravir (MOV; other names: MK-4482, EIDD-2801), a ribonucleoside analogue, has emerged as an effective SARS-CoV-2 treatment by inducing catastrophic viral mutagenesis during replication. However, there are growing concerns as MOV’s potential to induce host DNA mutagenesis remains an open question. Analysis of RNA-seq data from SARS-CoV-2–infected MOV-treated golden hamster lung biopsies confirmed MOV’s efficiency in stopping SARS-CoV-2 replication. Importantly, MOV treatment did not increase mutations in the host lung cells. This finding calls for additional mutation calls on host biopsies from more proliferative tissues to fully explore MOV’s hypothesized mutagenic risk.

## Introduction

Despite the efficiency of vaccines in combating COVID-19, the emergence and spread of SARS-CoV-2 variants of concern increases the threat of potential escape from “natural or vaccine-induced immunity.”^1^ Broad-spectrum preventive or treatment drug options offer an alternative in keeping up with SARS-CoV-2 evolution.^2^ Molnupiravir (MOV) is an oral antiviral medication that inhibits SARS-CoV-2 replication through viral RNA mutation buildup.^2-11^ Unlike other ribonucleoside analogue antiviral agents, key to MOV’s efficiency in inducing catastrophic viral mutagenesis is its ability to evade SARS-CoV-2 exonuclease proofreading activity.^8^ MOV has shown promising phase 3 MOVe-OUT clinical trial results where early treatment “reduced the risk of hospitalization or death in at-risk, unvaccinated adults with Covid-19.”^12,13^ Importantly, MOV was effective against all three SARS-CoV-2 variants of concern (delta, gamma, and mu) which were tested.^12,13^

Despite the encouraging results, there are growing concerns for potential host cell’s mutagenesis since MOV’s active metabolite, β-D-N^4^-hydroxycytidine (NHC), can be metabolized to 2′-deoxyribonucleotide and theoretically incorporated into the host genome. A recent in vitro hypoxanthine phosphoribosyltransferase (*HPRT*) mutation assay in Chinese hamster ovary (CHO-K1) cells suggested potential NHC-induced mutations.^5^ Indeed, their RNA sequencing of *HPRT* gene showed missense substitution and frame shifts mutations.^5^ Given the lack of in vitro or in vivo whole genome/exome sequences for host cells which have been exposed to MOV, potential global host DNA mutagenesis remains an open question. Here, publicly available RNA-seq data were used as a surrogate for probing host DNA mutations as this would show up in the resultant mRNA transcripts. Results confirm MOV’s efficiency in SARS-CoV-2 elimination. Importantly, there was no increase in mutational load in host cells lung biopsy. I also offer a perspective on additional tests that would help in assuring the scientific community and general public of MOV’s safety.

## Methods

### RNA-seq reads processing, alignment, and mutation calls

Lung biopsy samples (GSE168095; see Table 1) paired-end RNA-seq reads (fastq format) were downloaded from NCBI’s Sequence Read Archive (SRA).^2^ They included uninfected (n = 2), infected vehicle-treated (n = 4), and infected MOV (MK-4482, EIDD-2801) treated (n = 4).

**Table 1.** GSE168095 dataset samples used in this study.

GEO sample accession number	SRA sample accession number	Infection	Condition
GSM5128903	SRR13833624	Infected	EIDD-2801 treated
GSM5128904	SRR13833625	Infected	EIDD-2801 treated
GSM5128905	SRR13833626	Infected	EIDD-2801 treated
GSM5128906	SRR13833627	Infected	EIDD-2801 treated
GSM5128907	SRR13833628	Infected	Vehicle treated
GSM5128908	SRR13833629	Infected	Vehicle treated
GSM5128909	SRR13833630	Infected	Vehicle treated
GSM5128910	SRR13833631	Infected	Vehicle treated
GSM5128911	SRR13833632	Uninfected	Untreated
GSM5128912	SRR13833633	Uninfected	Untreated

SRA, Sequence Read Archive.

Reads were preprocessed with fastp (version 0.23.1)^14^ and subsequently split simultaneously into reads mapping golden hamster reference genome (MesAur1.0) and SARS-CoV-2 reference genome (NC_045512.2) using BBsplit function in BBmap (version 38.86).^15^ This approach has been successfully used on SARS-CoV-2 before.^4^ The splice aware aligner, STAR (version 2.7.9a),^16^ was used to align the dichotomized reads to their respective genome. Mutation calls was done on the resultant aligned MesAur1.0 “*.bam*” files using Strelka2 (version 2.9.2)^17^ which was recently shown to be optimal for variant calling even in low-read-depth single cells RNA-seq data.^18^ The *–rna* flag option was used to activate experimental settings for RNA-seq data variant calling. Only confident calls (mutations with a “PASS” filter flag) were considered for downstream analysis. BCFtools stats^19^ was used to extract out the statistics of the mutations called. SnpEff^20^ was used to annotate the mutations called and extract out missense and frameshift variants. Annotated VCF files are deposited here https://github.com/maringa780/Molnupiravir-VCF.

## Results

### MOV efficiently eliminates SARS-CoV-2 in host lungs

**Figure 1.**
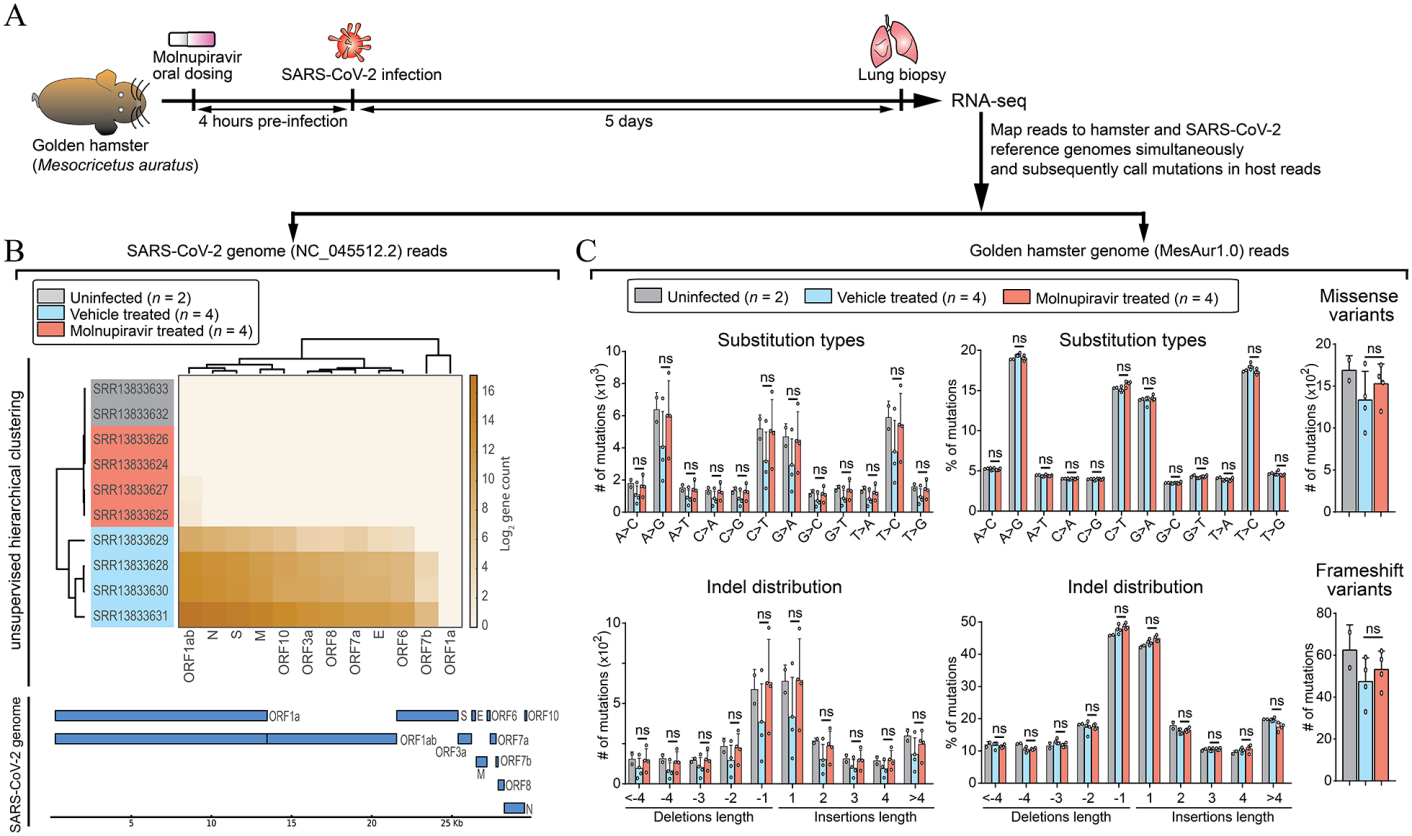
Molnupiravir (MOV) eliminates SARS-CoV-2 and does not induce mutations in host lungs. (A) Experimental setup (see the study by Bakowski *et al*^2^) for uninfected, MOV-, or vehicle-treated SARS-CoV-2–infected golden hamsters. RNA-seq reads were mapped to golden hamster (MesAur1.0) and SARS-CoV-2 (NC_045512.2) genomes simultaneously (BBsplit function in BBmap). (B) Top: Heatmap for unsupervised hierarchical clustering of read count mapping SARS-CoV-2 genome. Row names indicate accession codes used to download RNA-seq reads from NCBI’s Sequence Read Archive (SRA) database. Column names highlight SARS-CoV-2 gene transcripts. Bottom: Gene track highlighting SARS-CoV-2 transcripts location in the genome. (C) Top row: bar plots on number and percentage distribution of substitution mutations per sample. Resultant missense variants are included. Bottom row: bar plots on number and percentage distribution of indel length. Deletions and insertions are shown as negative and positive length, respectively. Percentage of deletions and insertions was computed separately. Resultant frameshift variants are included. All bar plots show mean ± SD. Statistical analysis (*t* test) was done on vehicle-treated versus MOV-treated samples. Uninfected samples are included to highlight the expected baseline mutations. ns: not significant.

To date, the study by Bakowski *et al*^2^ is the only one with publicly available RNA-seq data on MOV-exposed host biopsy (GSE168095). Briefly, the authors orally administered MOV or vehicle to golden hamsters, 4 hours prior to intranasal infection with SARS-CoV-2 (Figure 1A). They also included untreated/uninfected control animals (see Table1). Lung biopsies were collected 5 days post infection and analyzed using RNA-seq platform. In the current analysis, RNA-seq reads were downloaded, processed for quality control, and simultaneously mapped to golden hamster (MesAur1.0) and SARS-CoV-2 (NC_045512.2) genome (see section “Methods”). 98.5 ± 1.2% and 1.5 ± 1.2% (mean ± SEM) of reads mapped hamster and SARS-CoV-2 genomes, respectively, in untreated SARS-CoV-2–infected animals, a range observed earlier.^4^ In contrast, 99.999979 ± 0.0000079% and 0.000021 ± 0.0000079% of reads mapped hamster and SARS-CoV-2 genomes, respectively, in MOV-treated SARS-CoV-2–infected animals, indicative of MOV’s ability to diminish SARS-CoV-2 RNA replication. In line with this, SARS-CoV-2–infected MOV-treated samples were indistinguishable from uninfected samples in unsupervised hierarchical clustering (Figure 1B). In contrast, infected vehicle-treated samples had detectable high levels of SARS-CoV-2 gene count (Figure 1B). This is consistent with previous studies showing MOV’s ability to block SARS-CoV-2 replication.^2-11^

### No signs of MOV-induced mutagenesis in host lung cells

Next, mutation calls for reads mapping to golden hamster genome was done using appropriate parameters for RNA-seq platform data. Filtering was done to only compare high confident mutational calls (see section “Methods”). There was no significant difference in absolute numbers and percentile distribution of both substitution and indel mutations (Figure 1C). In addition, the substitutions and indels did not significantly change the number of gene missense and frameshift variants, respectively (Figure 1C). Taken together, there was no differential mutation enrichment under MOV treatment.

## Discussion

While MOV’s clinical trials have been promising, it is imperative that all possible risks are fully assessed to enable objective risk-benefit analysis decisions. The mutation analysis described here shows no signs of increased mutations in MOV-treated host lung biopsies. However, cell turnover/proliferation, a key requirement for mutagenesis induction, could be a confounding factor since it is quite low in the lungs.^21^ Potential genotoxicity of MOV has been addressed in part by Merck (Pharmaceutical company developing MOV) through in vivo Pig-a mutation assay, and Big Blue (cII Locus) transgenic assay in rats.^10^ In both assays, there was no difference in mutation rates observed between untreated and MOV-treated animals.^10^ This is in line with the current result showing lack of additional mutations in the host lung cells. While details of animal tissue used for the Big Blue assay have not been published, Pig-a assay uses blood samples,^22^ serving as an excellent sample source since blood cells have some of the highest cell turnover.^21^ It is worth noting that in vivo Pig-a assay had 82.4% sensitivity in a recent analysis of in vivo genotoxicity assays in detecting human carcinogens.^23^ Combining it with in vivo micronucleus assay was shown to improve the sensitivity to 94.1%.^23^ Following this recommendation, Merck did “in vitro micronucleus (with and without metabolic activation) and in vivo rat micronucleus assays,” with both assays showing no MOV-induced chromosomal damage.^10^ Nevertheless, the conclusion that “MOV is not considered to pose an increased risk of genotoxicity in clinical use”^10^ has been questioned by other researchers owing to lack of details on protocol used and assay sensitivity.^24,25^ While NHC (MOV’s active metabolite) showed positive mutagenesis in Zhou *et al*^5^ in vitro *HPRT* genotoxicity assay, the cells were cultured in the presence of NHC for 32 days in contrast to the typical 5 days of MOV treatment in animal studies^2^ and clinical trials.^12,13,26^ Interestingly, cells exposed to 1 minute of UV light showed higher (~1.3-fold to ~4.4-fold) inferred mutation effect compared to NHC’s highest effect in the *HPRT* genotoxicity assay.^5^ Given the contradicting results between Merck and Zhou *et al* read-outs of mutagenesis assays,^5,10^ whole genome/exome deep sequencing of highly proliferative host cells or tissues under MOV exposure would provide an unbiased broader perspective on global mutational differences if any. The necessity for this cannot be overstated given the potential implications of genotoxicity in initiating cancer or birth defects.

With mounting concerns on MOV’s safety being expressed within the scientific community and in mainstream media,^27^ more publicly available scientific data on MOV’s short-term or long-term genotoxic effect are needed to address legitimate questions raised. No matter what the data will show, this approach will strengthen public trust in the scientific community and the process of testing all available evidence to ensure public safety. In the meantime, MOV’s use should be restricted to COVID-19 patients with risk factor(s) for developing severe disease as its benefits would outweigh the hypothesized mutagenic risk.
